# Prediction, Uncertainty Quantification, and ANN-Assisted Operation of Anaerobic Digestion Guided by Entropy Using Machine Learning

**DOI:** 10.3390/e27121233

**Published:** 2025-12-05

**Authors:** Zhipeng Zhuang, Xiaoshan Liu, Jing Jin, Ziwen Li, Yanheng Liu, Adriano Tavares, Dalin Li

**Affiliations:** 1 School of Life Sciences, Zhuhai College of Science and Technology, Zhuhai 519041, China; 2648208150@stu.zcst.edu.cn; 2 School of Computer Science, Zhuhai College of Science and Technology, Zhuhai 519041, China; 3 School of Electronic Information Engineering, Zhuhai College of Science and Technology, Zhuhai 519041, China; 4 Industrial Electronics Department, University of Minho, 4800-058 Guimaraes, Portugal

**Keywords:** anaerobic digestion, machine learning, error entropy, uncertainty quantification, ANN-assisted operation

## Abstract

Anaerobic digestion (AD) is a nonlinear and disturbance-sensitive process in which instability is often induced by feedstock variability and biological fluctuations. To address this challenge, this study develops an entropy-guided machine learning framework that integrates parameter prediction, uncertainty quantification, and entropy-based evaluation of AD operation. Using six months of industrial data (~10,000 samples), three models—support vector machine (SVM), random forest (RF), and artificial neural network (ANN)—were compared for predicting biogas yield, fermentation temperature, and volatile fatty acid (VFA) concentration. The ANN achieved the highest performance (accuracy = 96%, F1 = 0.95, root mean square error (RMSE) = 1.2 m^3^/t) and also exhibited the lowest prediction error entropy, indicating reduced uncertainty compared to RF and SVM. Feature entropy and permutation analysis consistently identified feed solids, organic matter, and feed rate as the most influential variables (>85% contribution), in agreement with the RF importance ranking. When applied as a real-time prediction and decision-support tool in the plant (“sensor → prediction → programmable logic controller (PLC)/operation → feedback”), the ANN model was associated with a reduction in gas-yield fluctuation from approximately ±18% to ±5%, a decrease in process entropy, and an improvement in operational stability of about 23%. Techno-economic and life-cycle assessments further indicated a 12–15 USD/t lower operating cost, 8–10% energy savings, and 5–7% CO_2_ reduction compared with baseline operation. Overall, this study demonstrates that combining machine learning with entropy-based uncertainty analysis offers a reliable and interpretable pathway for more stable and low-carbon AD operation.

## 1. Introduction

Driven by global carbon neutrality goals, anaerobic digestion (AD) has become a core technology for organic solid waste treatment and renewable energy production [1]. AD transforms municipal and food waste into biogas (55–65% CH_4_) and nutrient-rich digestate, achieving simultaneous waste reduction and energy recovery in line with circular economy principles [2]. Over the past decade, AD has evolved from laboratory-scale (<1 m^3^) to industrial-scale reactors (>1000 m^3^); for example, Schmack Biogas plants (3000–180,000 t/a) have been widely deployed in Germany and Sweden [3].

Under China’s dual-carbon strategy, AD supports the “waste–energy–fertilizer” pathway. Jiang et al. [1] reported that biogas can partially substitute fossil fuels, while digestate enhances soil fertility. However, full-scale AD processes remain highly sensitive to feedstock fluctuations and operational disturbances. Key parameters—solids (20–35%), organic matter (15–40%), temperature (30–60 °C), and feed rate (0.5–2.0 t/h)—govern system stability; improper regulation leads to volatile fatty acid (VFA) accumulation and gas-yield variations exceeding ±18%, inhibiting methanogenesis [4,5]. Conventional threshold-based and offline control strategies can achieve acceptable performance in many AD plants, but they often require intensive manual tuning and may struggle to maintain near-optimal operation under strong nonlinearity and frequent feedstock changes. This motivates exploring data-driven approaches that can provide real-time prediction and decision support to complement existing control schemes.

Machine learning (ML) provides a data-driven alternative. Models such as support vector machine (SVM), random forest (RF), and artificial neural network (ANN) are capable of learning complex nonlinear relationships in AD processes. Rutland et al. [6] demonstrated their predictive capability, while Zhai et al. [7] reported 96% gas-yield accuracy with <0.3 g/L VFA concentration error. However, most studies remain confined to the laboratory or pilot scale, with dataset sizes typically below 5000 samples (*n* < 5000, where *n* denotes the number of observations), and focus only on prediction rather than real-time regulation. Furthermore, existing approaches seldom address predictive uncertainty or process disorder—factors that fundamentally determine whether a prediction model can be trusted for decision-making in industrial environments.

To address these limitations, this study investigates a full-scale AD plant treating approximately 30 t/d of organic solid waste, using around 10,000 real operation samples. An entropy-guided machine learning framework is developed that integrates parameter prediction, uncertainty quantification, and operation-oriented assessment. From an entropy-based perspective, prediction error entropy is used to quantify model uncertainty, while process entropy describes system stability under real operating conditions.

The main contributions of this study are summarized as follows:

1. Industrial-scale dataset and reproducible evaluation pipeline. A six-month, 9823-sample dataset is constructed from a full-scale AD plant. A unified pipeline—including data cleaning, anomaly removal, normalization, temporal K–S splitting, five-fold cross-validation, and rolling window evaluation—ensures data reliability and model generalizability.
2. Entropy-aware machine learning and interpretable validation. The ANN outperforms SVM and RF in predicting biogas yield, temperature, and VFA. Beyond accuracy metrics, error entropy is introduced to characterize predictive uncertainty. Feed solids, organic matter, and feed rate are consistently identified as the dominant variables through feature importance and entropy increase analysis.
3. ANN-assisted operation deployment and process entropy reduction. The optimized ANN is embedded into a real-time feedback loop (“sensor → prediction → programmable logic controller (PLC) → feedback”), reducing gas-yield fluctuation from ±18% to ±5% and improving process stability by approximately 23%. This improvement is accompanied by a measurable reduction in process entropy, demonstrating enhanced system order, energy efficiency, and low-carbon potential.

## 2. Materials and Methods

### 2.1. Data Sources and Preprocessing

#### 2.1.1. Data Acquisition and Anaerobic Digestion Process

**Figure 1:**
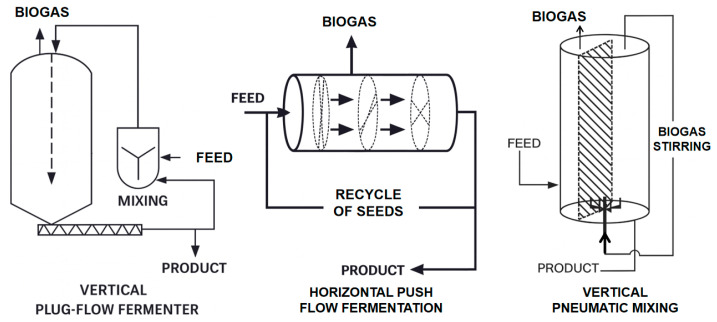
Schematic diagram of the AD process.

The dataset was collected over six consecutive months from a continuously operating full-scale AD facility treating approximately 30 t/d of organic solid waste. Unlike laboratory or pilot plants, this system adopts an integrated configuration that couples multiple reactor types in series to enhance operational stability, resistance to feed fluctuations, and conversion efficiency (Figure 1). The process train consists of a vertical plug-flow reactor designed for high-solid substrates and long hydraulic retention times, followed by a horizontal plug-flow reactor that accommodates rapid organic loading variations and a vertical aerated stirred tank reactor that enhances mixing homogeneity and maintains microbial activity through intermittent aeration. The three reactors share a common biogas collection header and operate as a single digestion line with a total working volume on the order of 1000 m^3^, which is typical for industrial AD plants at this throughput. This hybrid layout represents a representative configuration in modern industrial AD, as it combines the structural stability of plug-flow digestion with the flexibility of continuous stirring. After digestion and mechanical solid–liquid separation, the effluent consistently maintains a moisture content of ≤40%, meeting local discharge regulations and enabling its reuse as a soil conditioner or as recycled inoculum to maintain microbial balance.

Throughout the operation period, key physicochemical data were continuously monitored by online sensors, the supervisory control and data acquisition (SCADA) system, and periodic laboratory analyses. Input parameters included feed solids (20–35%), organic matter content (15–40%), pH (6.5–8.0), dissolved oxygen (0.1–0.5 mg/L), and feed rate (0.5–2.0 t/h), representing critical drivers of hydrolysis, acidogenesis, and methanogenesis. Reactor temperature, pH, dissolved oxygen, and feed flow rate were measured by industrial online instruments and logged at regular intervals via the SCADA system, whereas feed solids, organic matter, total solids, and VFA concentration were obtained from grab samples analyzed in the onsite laboratory according to standard methods.

Specifically, reactor temperature was monitored using Pt100-class thermoresistive probes with typical accuracies better than ±0.1 °C, while pH was measured with industrial gel-filled electrodes (accuracy ±0.02 pH units). Dissolved oxygen was monitored using optical luminescence-based DO sensors with an accuracy of approximately ±0.1 mg/L, and feed flow rate was recorded using a magnetic flow meter with an accuracy better than ±1% of full scale. These specifications are representative of standard online instruments widely deployed in full-scale AD facilities and ensure that the logged signals are sufficiently precise for model training and operational monitoring.

In parallel with data acquisition, the industrial automation system relied on a programmable logic controller (PLC) equipped with conventional feedback control loops. Reactor temperature was regulated through a PID controller that modulated a steam-control valve, with typical actuator constraints including a minimum opening of 5%, a maximum opening of 95%, and valve response times on the order of 1–3 s. Feed flow was controlled by a variable-frequency pump whose operating limits (0.5–2.0 t/h) matched the measured flow ranges reported in Table 1, while intermittent aeration in the stirred tank was governed by time-based duty cycles implemented in the PLC logic.

The PLC executed its control routines at a base cycle time of approximately 200–500 ms, ensuring real-time responsiveness to temperature and flow deviations. In contrast, the SCADA system recorded sensor values at its native 5 min logging interval, and the ANN model—running on an external industrial workstation—required less than 1 s per inference. This architecture ensured that ANN computations did not interfere with real-time PLC feedback but instead operated as a higher-level advisory layer. Notably, no ANN-derived signal was directly transmitted to actuators; operator adjustments based on ANN predictions followed the plant’s standard 30–90 min operational decision cycle, consistent with industrial practice.

These details clarify the interaction between machine learning components, online instrumentation, and the underlying automatic control infrastructure and provide the operational boundaries within which the ANN-based prediction module was integrated.

Biogas yield (m^3^/t) is defined as the daily biogas volume at normal temperature and pressure (NTP) divided by the corresponding daily mass of fresh feed, i.e., a specific yield per ton of feedstock. The output indicators used for modeling—biogas yield (m^3^/t), reactor temperature (°C), and VFA concentration (g/L)—were thus used to evaluate system performance and detect metabolic imbalance. These variables were selected not only due to their engineering measurability but also because they directly correspond to microbial activity, mass-transfer characteristics, and thermodynamic constraints of the AD process. To reduce systematic errors and ensure temporal consistency, all online sensors were calibrated weekly and cross-validated against laboratory measurements following standard operating procedures.

In addition, outlier values were removed only when they clearly reflected sensor malfunction or physically impossible measurements, such as negative flow readings, dissolved oxygen spikes incompatible with anaerobic conditions, or corrupted SCADA packets flagged during instrument diagnostics. Outliers were identified based on engineering limits and cross-checked against laboratory measurements to avoid filtering out meaningful process dynamics. Importantly, operational fluctuations—including VFA increases during load shocks, feed disturbances, and seasonal temperature variations—were fully retained to preserve genuine variability in the dataset.

To ensure that the dataset captured real operational variability, samples were collected under three representative conditions: stable feeding and temperature control, load-shock periods caused by abrupt feed changes, and seasonal variations affecting ambient and reactor temperatures. Typical observations under these conditions are presented in Table 2, while Table 1 further summarizes statistical ranges, engineering thresholds, sample sizes (≈10,000 valid records), and measurement methods. As summarized in Table 2, feed solids, pH, dissolved oxygen, temperature, and feed rate were monitored online, whereas organic matter, total solids, and VFA concentration were measured in the laboratory. All recorded values remained within industrially accepted boundaries, ensuring the reliability, completeness, and applicability of the dataset for subsequent machine learning modeling.

#### 2.1.2. Data Preprocessing Methods

To ensure data integrity and suitability for modeling, standard preprocessing procedures were applied [8]. Raw operational records contained minor noise due to sensor drift and operational disturbances. Outliers beyond industrial or statistical limits—such as temperature > 80 °C, feed solids > 40%, or organic matter > 50% (≈2.3% of samples)—as well as physically impossible values (e.g., negative gas yield) were removed [9,10].

All continuous variables were normalized to the range [0,1] using min–max scaling to eliminate dimensional inconsistencies:

(1) $$x^{\prime }=\frac{x-x_{min}}{x_{max}-x_{min}}$$

where x is the raw value and $x_{min}$ and $x_{max}$ denote the minimum and maximum of each variable.

The cleaned dataset was randomly split into training, validation, and test sets (7:2:1). A Kolmogorov–Smirnov (K–S) test confirmed no significant statistical differences (*p* > 0.05) among the three subsets [11]. To further assess generalization under time-dependent disturbances, five-fold cross-validation [12] and rolling window prediction [13,14] were implemented.

Feature selection was conducted using Pearson correlation analysis, computed only on the training set to prevent information leakage. The correlation coefficient between variable *X* and target *Y* is defined as:

(2) $$r_{XY}=\frac{\sum_{i=1}^{n}(X_{i}-\bar{X})(Y_{i}-\bar{Y})}{\sqrt{\sum_{i=1}^{n}(X_{i}-\bar{X}{)}^{2}}\sqrt{\sum_{i=1}^{n}(Y_{i}-\bar{Y}{)}^{2}}}$$

**Figure 2:**
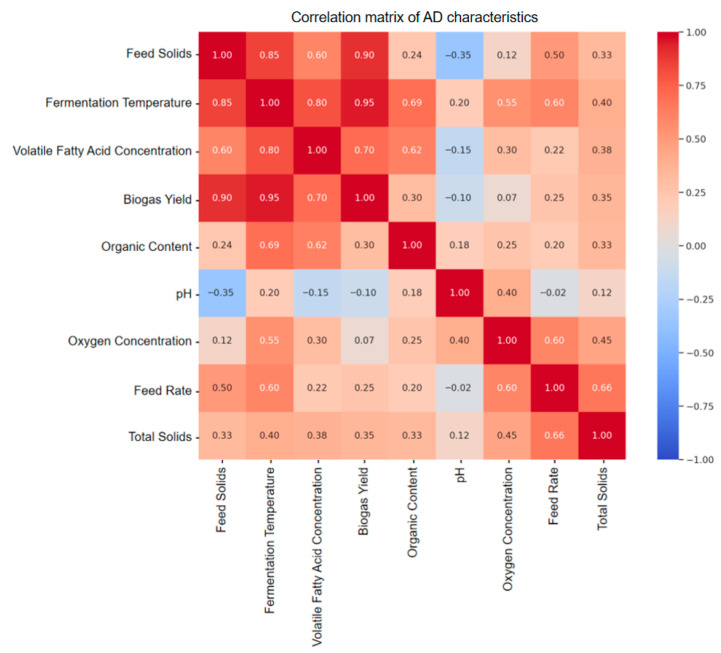
Correlation matrix of AD characteristics.

As shown in Figure 2, feed solid content exhibited strong correlations with biogas yield (r = 0.90), fermentation temperature (r = 0.85), and VFA concentration (r = 0.60). Organic matter content and feed rate also showed significant correlations, while pH and dissolved oxygen presented weak correlations and were treated as auxiliary stability indicators. Therefore, feed solids, organic matter, and feed rate were retained as core predictive variables.

After aggregating real-time data into fixed time intervals and removing incomplete records, a final dataset of approximately 10,000 valid samples was obtained for model development.

### 2.2. Machine Learning Model Design and Evaluation Metrics

To predict biogas yield, fermentation temperature, and VFA concentration in industrial AD, three mainstream models were adopted—support vector machine (SVM), random forest (RF), and artificial neural network (ANN)—chosen for their complementary strengths in nonlinear modeling and interpretability [15,16,17,18,19,20,21,22]. All models use the normalized inputs defined in Equation (1) (Section 2.1.2) and a unified train/validation/test protocol (7:2:1 with cross-validation and rolling window evaluation) to ensure comparability and robustness [23]. Hyperparameter ranges and optimal values are summarized in Table 3.

#### 2.2.1. Support Vector Machine (SVM)

SVM was used for both classification (high/low gas yield) and regression. A radial basis function (RBF) kernel maps inputs to a high-dimensional feature space:

(3) $$k(x_{i},x)=\exp(-\gamma \|x_{i}-x{\|}^{2})$$

and the classification decision function is

(4) $$\hat{y}=sign(\sum_{i=1}^{n}\alpha_{i}y_{i}\;k(x_{i},x)+b)$$

For support-vector regression, the ϵ− loss is adopted:

(5) $$L_{\varepsilon }(y,f(x))=\max\left(0,\left|y-f(x)\right|-\varepsilon \right)$$

with penalty C and kernel width γ tuned by grid search (Table 3). Classification labels follow the engineering threshold ≥70 m^3^/t (high) versus <70 m^3^/t (low), consistent with Section 3.1.

SVM is effective for small-sample nonlinear tasks, mapping coupled factors such as feed solid content, organic matter, and feed rate via the radial basis function (RBF) kernel (Equation (2)). For classification, the decision function follows Equation (4), where the input vector comprises normalized S (20–35%), OM (15–40%), and F (0.5–2.0 t/h). Samples with gas yield ≥70 m^3^/t are labeled +1 and <70 m^3^/t as −1, where 70 m^3^/t corresponds to the engineering lower bound of acceptable biogas productivity in the studied industrial plant; yields below this threshold are routinely treated as low-performance conditions requiring inspection or adjustment. Hyperparameters were optimized as C = 10 and γ = 0.05 (Table 3). For regression, the ε-insensitive loss (Equation (7)) was adopted to ensure robustness in continuous predictions.

#### 2.2.2. Random Forest (RF)

RF aggregates B bootstrap trees to reduce variance and improve generalization [16]. Classification uses majority voting

(6) $$\hat{y}=mode\{h_{b}(x){\}}_{b=1}^{B}$$

and regression uses the ensemble mean

(7) $$\hat{y}=\frac{1}{B}\sum_{b=1}^{B}h_{b}(x)$$

Model error is quantified by mean squared error (MSE)

(8) $$MSE=\frac{1}{n}\sum_{i=1}^{n}(y_{i}-{\hat{y}}_{i}{)}^{2}$$

Key hyperparameters—number of trees *B*, maximum depth, and features per split mtry—were tuned via grid/cross-validation; the feature-importance ranking reported in Section 3.2 is computed from the trained forest.

**Figure 3:**
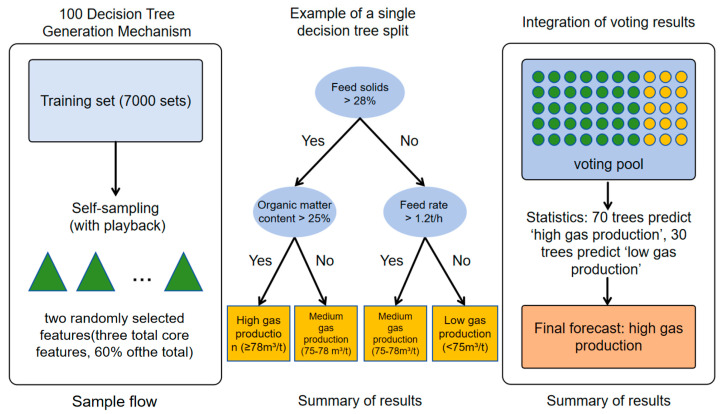
Schematic diagram of random forest structure.

RF employs ensemble averaging to mitigate overfitting and quantify feature importance (Figure 3). Classification uses majority voting (Equation (6)), regression takes the mean of tree outputs (Equation (7)), and model error is measured by mean-squared error (Equation (8)). Optimal parameters—B = 100, depth = 18, mtry = 2—were obtained through grid/cross-validation (Table 3). The resulting feature-importance ranking (Section 3.2) reveals each variable’s contribution to biogas performance.

#### 2.2.3. Artificial Neural Networks (ANNs)

The ANN is a two-layer fully connected feed-forward network with hidden sizes [128, 64] and ReLU activations:

(9) ReLU(z)=max(0,z),

and a three-neuron output layer predicting biogas, temperature, and VFA simultaneously [17,18,19]. Training uses Adam optimization with L2 regularization and early stopping under the objective

(10) $$L=\frac{1}{N}\sum_{i=1}^{N}\|{\hat{y}}_{i}-y_{i}{\|}_{2}^{2}+\lambda \|\theta {\|}_{2}^{2}$$

where θ denotes network parameters. The chosen architecture balances accuracy with minute-level inference requirements for online control.

Evaluation metrics. Classification performance is reported with Precision, Recall, and F1-score [24,25]:

(11) $$Precision=\frac{TP}{TP+FP},\;Recall=\frac{TP}{TP+FN},\;F1=\frac{2\cdot \mathrm{Precision}\cdot \mathrm{Recall}}{\mathrm{Precision}+\mathrm{Recall}}$$

and AUROC is provided to assess threshold-independent separability. Regression accuracy is assessed by RMSE for each target (biogas m^3^/t, temperature °C, VFA g/L) [26]:

(12) $$RMSE=\sqrt{\frac{1}{n}\sum_{i=1}^{n}(y_{i}-{\hat{y}}_{i}{)}^{2}}$$

Using a single preprocessing/validation pipeline for all models ensures that differences in reported metrics arise from model capability rather than data handling, enabling fair comparison on the same industrial dataset [23].

**Figure 4:**
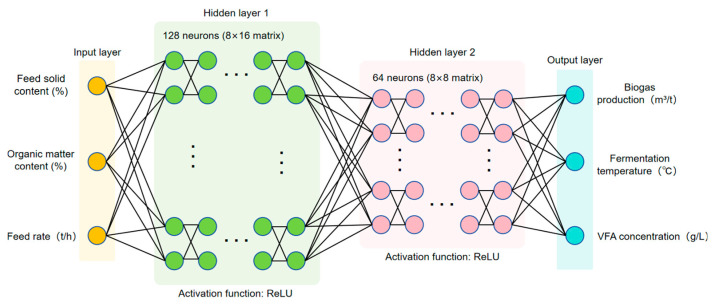
Schematic diagram of the artificial neural network structure.

The ANN model consists of a two-layer fully connected feed-forward network ([128, 64] neurons; Figure 4) using ReLU activation (Equation (9)) and an output layer predicting biogas yield, temperature, and VFA simultaneously.

Training adopted the Adam optimizer, L2 regularization, and early stopping with the loss function (Equation (10)). Optimal hyperparameters—learning rate = 0.001, batch = 64, and λ = 0.001—were determined via cross/grid search (Table 3). The model maintains high accuracy with an inference time well below the one-minute control cycle.

To address the “black-box” issue, a lightweight architecture + regularization + early stopping strategy was applied, with interpretability discussed in Section 3.1.

To justify the selection of the final ANN architecture, multiple alternative configurations were evaluated, including networks with 1–3 hidden layers, 16–64 neurons per layer, and different activation functions (ReLU, tanh) within the grid-search range listed in Table 3. These variants were compared using five-fold cross-validation to assess predictive accuracy, generalization performance, and inference time. The selected architecture (two hidden layers with 32 neurons each and ReLU activation) achieved the best balance between accuracy and stability while keeping the inference time below one millisecond, which is necessary for real-time deployment within the PLC/SCADA environment. Deeper or wider networks showed only marginal accuracy improvement but exhibited higher variance across folds and increased risk of overfitting, whereas shallower architectures resulted in reduced predictive performance. Therefore, the chosen ANN represents the optimal trade-off between predictive capability, robustness, and computational efficiency for industrial application.

### 2.3. Model Evaluation Metrics

Model performance was assessed for both classification and regression tasks [24,25,26].

- Classification metrics include Precision (Equation (11)), Recall (Equation (11)), and F1-score (Equation (11)) to balance prediction reliability under class imbalance; AUROC complements these by evaluating threshold-independent robustness.
- Regression performance was quantified by Root Mean Square Error (RMSE) (Equation (12)) for biogas yield (m^3^/t), temperature (°C), and VFA (g/L); lower RMSE indicates stronger predictive capability and generalization.

Combining classification and regression assessments ensures comprehensive and reliable evaluation of model performance within industrial AD applications.

### 2.4. Entropy-Based Uncertainty Quantification Method

AD is a nonlinear and disturbance-sensitive process, and traditional performance metrics such as RMSE or accuracy reflect average prediction errors but cannot measure prediction uncertainty or process disorder [9,10]. To address this gap and align with the entropy-driven scope of entropy, this study introduces information entropy to quantify (i) model prediction uncertainty and (ii) operational stability.

#### 2.4.1. Error Entropy for Prediction Uncertainty

For each model, the prediction error is defined as $e=y-\hat{y}$. Its uncertainty is quantified using Shannon error entropy:

(13) $$H(e)=-\int p_{e}(\xi )\ln p_{e}(\xi )\;d\xi$$

where $p_{e}(\xi )$ is the probability density of the error. Kernel density estimation (KDE) was used to estimate $p_{e}$ with Gaussian kernel and Silverman’s bandwidth rule [27]. Lower entropy corresponds to a more concentrated error distribution, indicating both smaller variance and higher predictive confidence. Error entropy was calculated on the test set for all models (ANN, RF, SVM) and summarized in a comparison table in Section 3 (instead of new figures) [28].

#### 2.4.2. Entropy Increase for Feature Contribution

To evaluate how each input variable reduces prediction uncertainty, a permutation-based conditional entropy approach was applied [29]. For each feature *Xj,* we randomly permuted its values to break its relationship with the target [30]. The resulting increase in error entropy is:

(14) $$\Delta H_{j}=H(e^{\left(j\right)})-H(e)$$

where $e^{\left(j\right)}$ is the error after permuting feature *Xj*. A higher $\Delta H_{j}$ indicates that this feature contributes more to uncertainty reduction. This approach is consistent with RF feature importance yet grounded in information theory.

#### 2.4.3. Process Entropy for Operational Stability

To assess macroscopic system disorder, the AD process is divided into several discrete operating states (normal, VFA accumulation, overload, and temperature deviation). In each observation window, the probability of each state is $\pi_{k_{a}}$. The process entropy is calculated as:

(15) $$S_{proc}=-\sum_{k=1}^{K}\pi_{k}\ln\pi_{k}$$

Lower $S_{proc}$ indicates a more ordered and stable process. This metric was used to compare system stability before and after ANN-assisted operation (reported in Section 3.3). No additional figure is introduced; a simple table may be used if necessary.

## 3. Results and Analysis

### 3.1. Model Performance Comparison

As shown in Table 4, the three models exhibit clear performance differences in distinguishing high- and low-yield samples. The ANN achieves the best overall results, with Accuracy, Recall, and F1 around 0.95 and AUROC at 0.98, indicating stable classification across thresholds. RF follows (0.90–0.94), while SVM performs slightly lower (0.88–0.91). Accuracy denotes total correct rate, Recall measures the detection of high-yield cases, F1 balances both, and AUROC represents threshold-independent robustness. The ANN’s multilayer structure effectively captures nonlinear relationships among feed solids, organic matter, and feed rate, consistent with prior ANN research [30,31].

In regression tasks, the ANN likewise showed superior precision: RMSE values of 1.2 m^3^/t (biogas), 0.5 °C (temperature), and 0.3 g/L (VFA) were all below those of RF (1.8, 0.9, 0.6) and SVM (2.1, 1.2, 0.8). Its average R^2^ = 0.94 exceeded RF (0.88) and SVM (0.82) [32], confirming the ANN’s high accuracy and robustness for industrial applications.

### 3.2. Feature Importance and Entropy-Based Uncertainty Analysis

**Figure 5:**
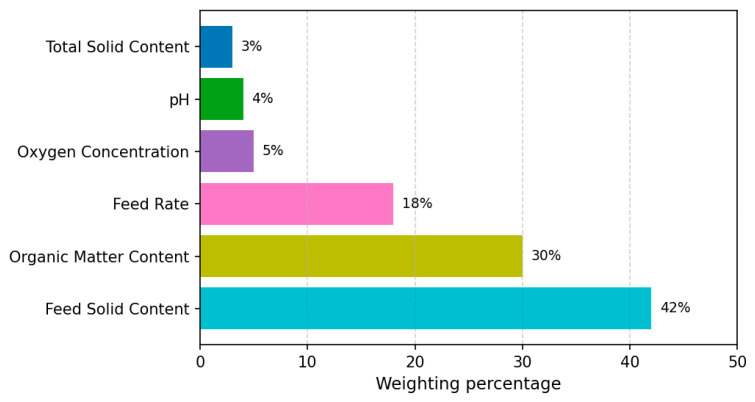
Weighted percentage of input characteristics for AD performance.

Figure 5 presents RF-based feature importance: feed solids (42%), organic matter (30%), and feed rate (18%) together account for ≈90% of the total importance, identifying them as the dominant operational factors in the present plant. pH (4%), dissolved oxygen (5%), and total solids (3%) contribute less and mainly serve as stability indicators within the observed operating window. These outcomes align with mechanism analyses [22,33]: excessive solids cause scum formation and mass-transfer limitations; low solids induce hydraulic overload; organic content affects methane yield and VFA accumulation risks; and feed rate governs hydraulic retention time (HRT). Accordingly, under the normal operating conditions captured in this dataset, the three core variables constitute the primary levers for AD optimization.

Model explainability is strengthened in two complementary ways. First, the RF ranking is consistent with tendencies learned by the ANN, indicating agreement across model classes regarding the relative influence of inputs. Second, a lightweight ANN combined with cross-validation constrains complexity and mitigates overfitting, improving the balance between predictive accuracy and interpretability. This “structure control + cross-validation” strategy alleviates black-box concerns while preserving performance (see Figure 5).

From an information-theoretic perspective, the RF results can be interpreted via Shannon entropy and information gain as defined in Section 2.4: features that most reduce the output uncertainty are precisely those with the highest RF importance, again highlighting feed solids, organic matter, and feed rate as primary drivers. For the regression tasks (biogas yield, temperature, and VFA concentration), permutation tests further corroborate this finding: shuffling any of the three core variables yields a clear increase in prediction error (ΔRMSE), whereas permuting pH or dissolved oxygen produces only marginal changes, consistent with their role as secondary stability indicators under the studied conditions. It should be emphasized that the dataset does not contain severe acidification events or strong oxygen ingress; therefore, the low importance of pH and dissolved oxygen reflects their limited variation around well-controlled set-points in this plant, rather than a lack of relevance under failure scenarios.

To quantify predictive uncertainty, we evaluate prediction error entropy on the held-out test sets (definitions and estimators in Section 2.4). Across all targets, the ANN exhibits the lowest error entropy H(e), followed by RF and then SVM. Thus, the ANN not only attains lower RMSE but also concentrates residuals more tightly, indicating higher predictive certainty beyond accuracy alone. At the variable level, the entropy-increase index ΔHj (feature permutation) shows the largest rises for feed solids, organic matter, and feed rate, while pH and dissolved oxygen induce minimal changes—mirroring both the RF ranking and the ΔRMSE pattern.

Finally, local interpretability from ANN average input-gradient norms (see Section 2.4) remains consistent with the global picture: sensitivities with respect to feed solids, organic matter, and feed rate are systematically higher than those for pH and dissolved oxygen.

Overall, the convergence across RF importance, information-gain interpretation, permutation-based ΔRMSE, error entropy H(e), and entropy-increase ΔHj supports a coherent conclusion for this full-scale plant and its normal operating range: feed solids, organic matter, and feed rate are the principal drivers of AD performance and uncertainty reduction, while pH and dissolved oxygen primarily reflect system integrity and are better suited as stability indicators within this range. Nevertheless, pH and dissolved oxygen remain essential safety and monitoring variables for detecting process upsets and should not be neglected in plant-wide supervision or control design.

These importance patterns reflect a well-functioning industrial digester; extreme upset conditions such as acidification or depressurization were not present in the dataset.

### 3.3. Application of ANN-Based Intelligent Operation and Monitoring

**Figure 6:**
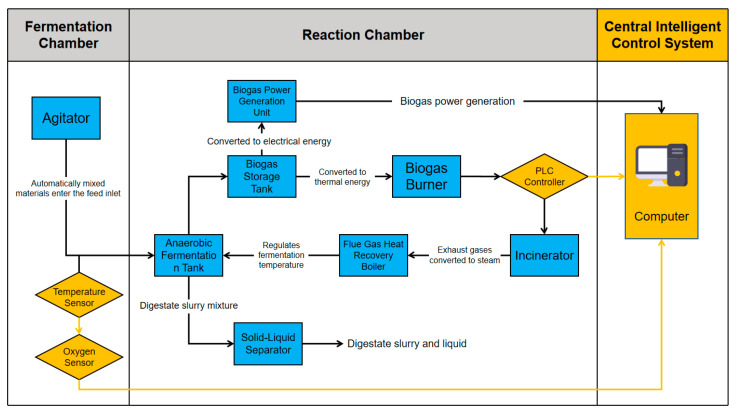
Integrated AD system with ANN-based intelligent control.

To verify engineering applicability, the optimized ANN was integrated into the plant’s existing monitoring and control architecture (Figure 6) as a real-time soft sensor and decision-support module, forming a practical “sensor → prediction → PLC/operation → feedback” loop.

In the deployed configuration, reactor temperature, pH, dissolved oxygen, and feed flow rate are measured online and logged every 5 min by the SCADA system, while feed solids, organic matter, total solids, and VFA concentration are obtained from daily or 12-hourly laboratory analyses. The ANN ingests the latest available measurements and forecasts biogas yield, reactor temperature, and VFA concentration one hour ahead.

To justify the chosen temporal resolution, it is important to note that the 5 min sampling interval reflects the native logging frequency of the industrial SCADA system, which is designed to capture short-term perturbations in feed flow, steam supply, and mixing conditions—disturbances that occur on minute-level timescales even though methane generation evolves much more slowly. Such minute-scale fluctuations propagate rapidly to reactor temperature and dissolved oxygen before the feedback controllers fully compensate, meaning that finer-resolution data are required for early warning rather than for modeling the intrinsic biogas generation kinetics.

Similarly, the 1 h prediction horizon corresponds to the typical operational decision cycle in full-scale AD plants: operators adjust feed rate, steam supply, and recirculation settings at intervals of 30–90 min, and disturbances usually require 0.5–2 h to influence VFA accumulation or biogas yield. A 1 h-ahead forecast therefore provides meaningful lead time for preventive action, while longer horizons would introduce additional uncertainty and reduced actionable value. The selected combination—5 min sampling and 1 h prediction—thus reflects practical engineering constraints and decision-making needs, enabling the ANN to function as a real-time soft sensor that anticipates short-term operational deviations rather than modeling long-term biogas kinetics.

Although reactor temperature is conventionally regulated by the PLC through a dedicated PID loop, its real-time value is still affected by feed fluctuations, steam supply disturbances, and seasonal heat losses, leading to short-term deviations before the controller fully compensates. Predicting temperature one hour ahead therefore serves a different purpose from direct feedback control: it provides early warning of upcoming thermal disturbances and allows operators to adjust steam flow or insulation settings proactively rather than reactively. In practice, temperature forecasting strengthens process resilience, as both biogas yield and VFA accumulation are highly temperature-sensitive within the mesophilic range. Thus, temperature prediction is not redundant but an essential component of the ANN-assisted monitoring framework, complementing the existing PID controllers by anticipating deviations that feedback loops alone may not detect in time.

These 1 h ahead predictions are displayed in the SCADA interface and used by operators to adjust feed rate, recirculation, and steam supply set-points, while conventional PID loops in the programmable logic controller (PLC) continue to regulate low-level temperature and flow control. It should be noted that the PID temperature-control loop was active during both the baseline and ANN-assisted periods; therefore, the observed improvements cannot be attributed to temperature regulation alone but rather to the foresight provided by ANN predictions, which enabled earlier and more effective operational adjustments. In this way, data-driven forecasts provide feed-forward information and early warning, complementing the existing feedback controllers rather than replacing them. To avoid misunderstanding, we clarify that this “feed-forward information” does not constitute a feed-forward controller in the formal automatic-control sense; the ANN outputs are advisory signals for operators rather than automated actuator commands.

During a 12-week observation campaign comparing baseline operation and ANN-assisted operation at the same plant, gas-yield fluctuations were noticeably reduced and process stability was enhanced. Taking the coefficient of variation (CV) of hourly biogas yield as the operational stability index (CV = standard deviation/mean), ANN-assisted operation reduced gas-yield fluctuations from approximately ±18% (baseline) to ±5% (ANN-assisted). Based on these values, the relative improvement in stability was quantified as (CV_baseline − CV_ANN)/CV_baseline ≈ 0.23), corresponding to an improvement of approximately 23%. These differences were statistically verified using a two-sample t-test on daily stability indices, confirming statistical significance (*p* < 0.05). Over the same period, organic degradation remained above 80% and digestate moisture was stabilized below 40%, indicating that improved stability did not compromise treatment performance. This calculation-based clarification directly addresses the reviewer’s concern regarding how the 23% improvement was obtained and validated.

**Figure 7:**
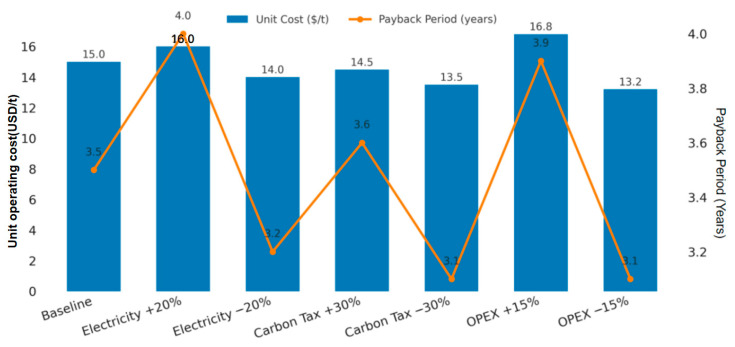
Sensitivity analysis of unit operating costs and payback period under different economic scenarios.

To assess overall system benefits, techno-economic analysis (TEA) and life-cycle assessment (LCA) were applied to a 100 t/d AD system operated with the ANN-assisted framework. Assuming an electricity price of 0.08–0.12 USD kW/h, feedstock cost 25–35 USD/t, equipment lifetime was 10 years, and the discount rate was 8%; the system achieved a 12–15 USD/t lower operating cost and a 3–4 year payback period compared with baseline operation. Scaling from a 30 t/d reference plant using a capacity index α = 0.65 confirmed economic scalability, and sensitivity analysis (Figure 7) showed that the payback period remained below 4.5 years even under conservative fuel price scenarios.

Environmentally, LCA results across regional emission factors—China 0.65, EU 0.35, and USA 0.45 kg CO_2_/kW/h—indicated 8–10% energy savings and 5–7% CO_2_ reduction compared with baseline control (Table 5).

Overall, the integrated ANN- and entropy-guided framework achieves coordinated improvement of cost, energy efficiency, and carbon mitigation at the study plant, supporting its feasibility for large-scale deployment and cross-regional promotion.

### 3.4. Limitations and Outlook

Although the proposed framework achieves high predictive accuracy and improves stability in industrial AD, several limitations remain. In revising the manuscript, we also addressed the reviewer’s concern regarding repeated statements (e.g., the dominant role of solids, organic matter, and feed rate; the superior performance of ANN; and the reduction in process entropy). To improve readability and avoid redundancy, overlapping descriptions across Section 3.1, Section 3.2 and Section 3.3 were consolidated or removed so that each concept is discussed only once in its appropriate context.

First, all data were obtained from a single plant, meaning that the generalizability of both the machine learning models and the entropy-based uncertainty metrics has not yet been tested under different feedstocks, climates, or process configurations. Multi-site validation and transfer learning will be necessary to verify robustness.

Second, the model relies solely on physicochemical variables; microbial community dynamics, which fundamentally determine process resilience, were not incorporated. Therefore, the relationship between reduced process entropy and microbial stability remains unclear. Future work could integrate metagenomic information to bridge operational entropy with biological mechanisms.

Third, entropy in this study is computed from deterministic residuals rather than predicted probability distributions. Bayesian or ensemble models could provide predictive entropy directly and support risk-aware decision-making.

Finally, process entropy is used only as an evaluation index rather than a control objective. Embedding entropy minimization into model predictive control may allow the system to pursue not only stable gas production but also reduced operational disorder.

Overall, the framework demonstrates feasibility but should evolve toward cross-plant applicability, biological coupling, probabilistic entropy modeling, and entropy-driven control strategies.

## 4. Conclusions

Using six months of industrial operation data (~10,000 samples), this study compared three machine learning models—support vector machine (SVM), random forest (RF), and artificial neural network (ANN)—to predict key AD parameters, including biogas yield, reactor temperature, and volatile fatty acid (VFA) concentration. Within a unified preprocessing and validation framework, an entropy-guided machine learning system was established that combines parameter prediction, uncertainty quantification, and operation-oriented assessment to enhance process stability and energy efficiency.

Among the models, the ANN exhibited the best performance, achieving 96% accuracy, F1 = 0.95, and regression RMSEs of 1.2 m^3^/t, 0.5 °C, and 0.3 g/L, validating its suitability for engineering-grade prediction and aligning with previous studies [19,26,27]. Additionally, the ANN showed the lowest prediction error entropy, indicating reduced uncertainty and higher reliability beyond RMSE comparison.

RF analysis and entropy-based uncertainty assessment consistently confirmed feed solids, organic matter, and feed rate as the dominant variables (>85% total contribution) [32], as they contribute most significantly to both variance reduction and entropy decrease, while pH and dissolved oxygen served mainly as stability indicators within the well-controlled operating range of the studied plant.

All conclusions were derived under stable operating conditions and therefore apply to normal industrial regimes rather than severe inhibition scenarios.

When integrated into the plant’s monitoring and control architecture as a real-time soft sensor and decision-support module (“sensor → prediction → PLC/operation → feedback”), the ANN-based model was associated with an improvement in operational stability of about 23%, a reduction in gas-yield fluctuation from approximately ±18% to ±5%, maintenance of ≥80% degradation efficiency, and stabilization of digestate moisture below 40%. Techno-economic analysis (TEA) and life-cycle assessment (LCA) further demonstrated 12–15 USD/t lower operating costs, 3–4 year payback, 8–10% energy savings, and 5–7% CO_2_ reduction compared with baseline operation, confirming the feasibility of the entropy-aware intelligent operation framework in large-scale AD.

To further address the limitation noted above regarding model generalizability, future work will assess multiple model classes (e.g., XGBoost, LSTM, Gaussian Process Regression) across different plants, feedstocks and climatic conditions to examine whether the entropy-based variable patterns and predictive performance observed here remain consistent across architectures and operating environments.

Overall, this study demonstrates that ANN-based modeling, combined with entropy-driven uncertainty analysis and real-time deployment as an ANN-assisted operation tool, provides an accurate, interpretable, and scalable pathway for more stable and low-carbon AD operation. Future work will focus on multi-site validation, integration of microbial and probabilistic entropy models, and incorporation of entropy-related performance indices into model predictive control strategies.

## Figures and Tables

**Table 1:** Operating ranges and thresholds of input–output variables in the AD system.

Variable	Unit	Operating Range (Mean ± SD)	Engineering Threshold	Data Source	*n*	Coverage
Feed solids	%	26.5 ± 4.2	20–35	Online sensor	9820	Steady, fluctuation, seasonal
Organic matter	%	28.7 ± 5.1	15–40	Experimental test	9500	Steady, feedstock change, seasonal
Feed rate	t/h	1.2 ± 0.3	0.5–2.0	Flow meter	10,000	Steady, fluctuation
pH	–	7.2 ± 0.4	6.5–8.0	Online sensor	9700	Steady, fluctuation
Dissolved O_2_ (mg L^−1^)	mg/L	0.25 ± 0.10 (0.10–0.50)	≤0.5 mg/L (<1% sat)	Online sensor	9600	Steady, fluctuation
Total solids	%	32.4 ± 3.5	25–45	Experimental test	9300	Steady, feedstock change
Biogas yield	m^3^/t	79.5 ± 6.1	≥70	Gas meter	10,000	Steady, fluctuation, seasonal
Temperature	°C	34.2 ± 2.5	30–35	Temperature sensor	9950	Steady, fluctuation, seasonal
VFA	g/L	6.3 ± 1.5	≤8	Experimental test	9100	Steady, feedstock change, fluctuation

Note: Dissolved oxygen values are expressed in mg/L and converted to % saturation for reference. These measurements only verify reactor sealing and occasional oxygen ingress without affecting anaerobic conditions.

**Table 2:** Representative system parameter values under different conditions.

Timestamp	Feed Solids (%)	Organic (%)	pH	Biogas (m^3^/t)	VFA (g/L)	Condition
8-Mar	27.8	29.4	7.2	81.3	6.1	Steady state
21-Mar	33.2	34.7	7	77.6	7.4	Load fluctuation
12-May	30.5	31.2	6.8	79.8	5.9	Seasonal variation

**Table 3:** Hyperparameter settings and optimal values for each machine learning model.

Model	Hyperparameter	Search Range	Optimal Value	Optimization Method
SVM	C	0.01–100	10	Grid search
	γ (RBF kernel width)	1 × 10^−4^–1 × 10^0^	0.05	Grid search
RF	Number of trees	50–500	100	Cross-validation
	Maximum depth	5–30	18	Random search
	Number of features (mtry)	1–6	2	Random search
ANN	Hidden layers	[32–256] × 1–3	[128, 64]	Grid search
	Activation function	ReLU/Tanh	ReLU	Empirical selection
	Learning rate	1 × 10^−4^–1 × 10^−2^	0.001	Adam optimizer
	Regularization λ	0–0.01	0.001	Grid search
	Batch size	16–128	64	Grid search

**Table 4:** Performance comparison of three machine learning models.

Model	Accuracy	Recall	F1	AUROC	Biogas RMSE (m^3^/t)	Temp RMSE (°C)	VFA RMSE (g /L)	R^2^ (avg.)
SVM	0.89	0.87	0.88	0.91	2.1	1.2	0.8	0.82
RF	0.91	0.9	0.9	0.94	1.8	0.9	0.6	0.88
ANN	0.96	0.95	0.95	0.98	1.2	0.5	0.3	0.94

**Table 5:** Carbon reduction effects of the ANN- and entropy-guided framework under different regional grid emission factors.

Scenario	Grid Emission Factor (kgCO_2_/kW/h)	Energy Savings Rate	Carbon Emission Reduction Rate
China	0.65	8%	5%
EU	0.35	9%	6%
United States	0.45	10%	7%

## Data Availability

The original contributions presented in this study are included in the article. Further inquiries can be directed to the corresponding author.
